# Dominant Chemical Interactions Governing the Folding Mechanism of Oligopeptides

**DOI:** 10.3390/ijms25179586

**Published:** 2024-09-04

**Authors:** Michele Larocca, Giuseppe Floresta, Daniele Verderese, Agostino Cilibrizzi

**Affiliations:** 1 Istituto di Metodologie per l’Analisi Ambientale–Consiglio Nazionale delle Ricerche (CNR-IMAA), Contrada, Santa. Loja, 85050 Potenza, Italy; 2 Department of Drug and Health Sciences, University of Catania, Viale A. Doria 6, 95125 Catania, Italy; 3 Dipartimento di Scienze Economiche e Statistiche, Università di Salerno, via Giovanni Paolo II, 132, 84084 Salerno, Italy; 4 Institute of Pharmaceutical Science, King’s College London, Stamford Street, London SE1 9NH, UK; 5 Centre for Therapeutic Innovation, University of Bath, Bath BA2 7AY, UK

**Keywords:** folding mechanisms, main mechanical forces, dominant chemical interactions, dihedral angle calculations

## Abstract

The hydrophobic effect is the main factor that drives the folding of polypeptide chains. In this study, we have examined the influence of the hydrophobic effect in the context of the main mechanical forces approach, mainly in relation to the establishment of specific interplays, such as hydrophobic and CH–π cloud interactions. By adopting three oligopeptides as model systems to assess folding features, we demonstrate herein that these finely tuned interactions dominate over electrostatic interactions, including H-bonds and electrostatic attractions/repulsions. The folding mechanism analysed here demonstrates cooperation at the single-residue level, for which we propose the terminology of “single residues cooperative folding”. Overall, hydrophobic and CH–π cloud interactions produce the main output of the hydrophobic effect and govern the folding mechanism, as demonstrated in this study with small polypeptide chains, which in turn represent the main secondary structures in proteins.

## 1. Introduction

The folding mechanism of polypeptide chains has been recently described as the output of main mechanical forces (MMFs), which develop as the result of highly specific chemical interactions established amongst side chains of amino acid residues in close spatial proximity within a sequence [1,2,3,4]. Earlier MMFs methodology studies support the assumption that there is a finely tuned mechanism regulating how each single amino acid in a polypeptide chain sequence ‘encodes’ its three-dimensional (3D) native structure. In agreement with previously reported studies [5], this proposed mechanism specifically relies on the cooperativity amongst amino acid side chain residues, namely the formation of suitable inter-residue chemical interactions amongst the amino acid side chains, such as hydrophobic, H-bond, electrostatic attraction/repulsion, CH–π cloud and π–π (pi-stacking) interactions [1,2,3,4].

Although advancements continue to be reported in the literature, focusing on improved methods of analysis and prediction [6], the folding process is still far from a full understanding. The main factor that drives the folding of polypeptide chains is well-known as the hydrophobic effect [7,8,9,10]. In this study, this effect was considered determinant to maximize the chemical interactions mentioned above, by simultaneous effect of the solvent, through the formation of hydrophobic cores. In this context, the work presented herein aims to provide further insights on the analysis of protein folding by describing the dominant chemical interactions that rule the mechanism itself. By applying the MMFs method and calculations [1,2,3,4], three small polypeptide chains, namely chignolin (PDB code: 1uao) [11], Leu-enkephalin [12] and CCR5 ECL2 (PDB code: 2mzx) [13], were used as model systems for this investigation. In particular, the dihedral angles in these reference polypeptide chains were calculated using the recently reported MMFs approach [4]. Subsequently, the structures obtained via MMFs analysis were modelled using the Molefacture plug-in [14] in VMD [15] and compared to experimentally determined structures by superimposition (see details in Supplementary Materials File S1: paragraph 1. Determination of the internal contacts). Briefly, the MMF approach focuses on interpreting, ‘numerically’, the interactions amongst the side chains of neighbour residues that, based on the length of the side chain, may establish interactions up to a certain distance [1,2,3,4]. This space length was defined by us as “threshold distance” r_i_ (see calculation spreadsheets for details: Supplementary Materials Files S2–S4), i.e., the initial distance that is used for the calculations [2,4]. Once r_i_ is obtained, it is possible to calculate the Coulombian force acting through the interacting atoms in the side chains and, in turn, define the equation that reports their respective potential energy [1,2]. The potential energy equation contains a partial dihedral angle to be calculated, where for a partial dihedral angle we take into consideration the rotation obtained after each single interaction of the side chain of a residue with its neighbour interacting residues. Specifically, given the threshold distance, it is possible to decipher the number of interactions that a residue might establish with the closest neighbour residues, enabling the calculation of all the allowed partial dihedral angles, which can then be added up to obtain the total dihedral angle. This latter is then compared to the experimental value. Furthermore, the MMFs approach allows to infer the equation of the torque moment, which also contains the partial dihedral angle to be calculated. Thus, equalizing potential energy and torque moment, it is possible to calculate the desired partial dihedral angle for a specific interaction by using the arctangent of the angle [4] (see details on calculations, reported in the calculations spreadsheets: Supplementary Materials Files S2–S4).

Overall, by applying the MMFs approach, we achieved a high match between the predicted/experimental structures examined in this work, which was further improved through energy minimization using the web server Chiron [16]. By data analysis, it was found that hydrophobic interactions, as well as CH–π clouds, dominate over all other types of possible interactions. Based on the MMFs approach, these two types of interplays may be considered as direct outputs of the hydrophobic effect, being the most represented for the determination of partial dihedral angles, which are then used for the calculations of total dihedral angles. For each of the polypeptides adopted in this study, we have considered all the possible interactions that can occur in the residues of the side chains on the basis of both threshold distances and evident chemical interactions [4]. Furthermore, the interactions that were useful to calculate the dihedral angles (highlighted in black, in the Supporting Information spreadsheets, Supplementary Materials File S2; Supplementary Materials File S3; Supplementary Materials File S4) showed that the best match was obtained when the largest partial rotations (or partial dihedral angles) were considered.

## 2. Results

**Figure 1:**
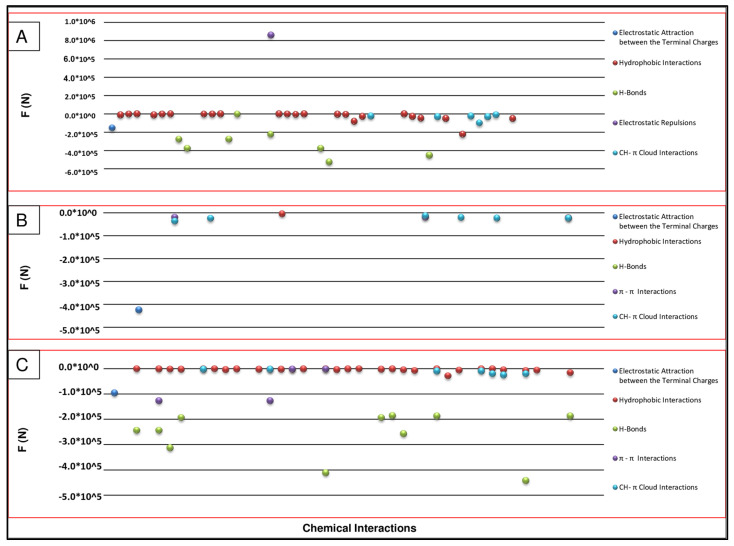
Mechanical forces related to (**A**) chemical interactions of the chignolin (1uao) folding process; (**B**) chemical interactions of the Leu–enkephalin folding process; and (**C**) chemical interactions of the CCR5 ECL2 (2mzx) folding process.

Primarily, the abovementioned evidence led to discovering that the most useful interactions are primarily the hydrophobic and CH–π cloud interactions, these being also the strongest interactions, as compared to H-bonds and electrostatic attractions (Figure 1), in agreement with the MMFs approach [1,2,3,4]. In contrast, pi stacking possessed an intermediate behaviour, while electrostatic repulsions were not represented much (Figure 1), even if these latter are the strongest of all the other types of interactions.

**Figure 2:**
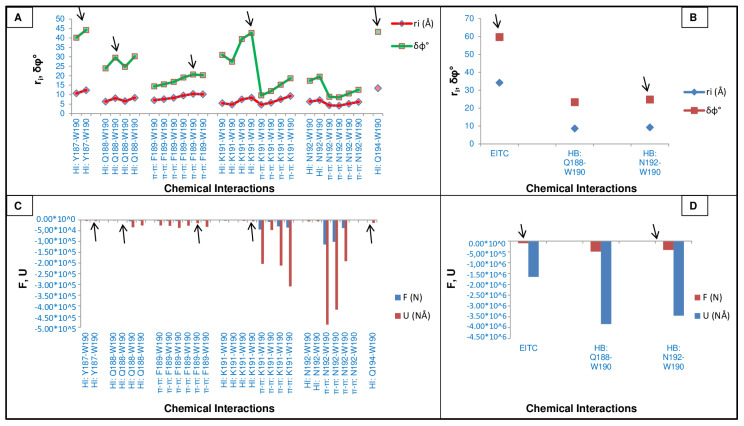
Physical measures that rule the folding mechanism at the single-residue level of TRP190. (**A**,**B**) Comparison between threshold distances and partial dihedral angles. (**C**,**D**) Comparison between developed mechanical forces and the related potential energy. Abbreviations: ri = threshold distance, δφ° = partial dihedral angle, F = mechanical force, U = potential energy, HI = hydrophobic interaction, HB = H-bond, EITC = electrostatic interaction between the terminal charges, π–π = π–π interaction or pi stacking, W = Tryptophan, Y = Tyrosine, Q = Glutamine, F = Phenylalanine, K = Lysine, N = Asparagine.

A single residue belonging to 2mzx (triptophan190 or TRP190) was also analysed in more detail, with the aim to further confirm these findings. We found that the folding mechanism of TRP190 relies on the establishment of several chemical interactions due to the chemical nature of the neighbour side chain residues. Specifically, TRP190 results involved hydrophobic, π–π, H-bond and electrostatic interactions between the two terminal charges, the latter being maintained constant for all the residues, in order to calculate accurately the related dihedral angles. As reported in Figure 2, there is the need to consider the largest values of the calculated partial dihedral angles for the calculation of the TRP190 dihedral angle φ. This confirms that the larger the rotations (partial dihedral angles), the higher the match with the experimental values. This feature implies that the larger rotation is provided by the longest threshold distance for the occurring chemical interaction. For all the possible interplays with each neighbour residue, the partial dihedral angle with the higher value of potential energy (and related mechanical force) is the one to take into consideration for the calculations, as shown in Figure 2 (i.e., black arrows) for the TRP190 interactions to achieve the calculation of the dihedral angle φ.

The approach applied to TRP190 was also used for all the amino acid residues in the three oligopeptides considered in this work. Notably, glycines express a variable behaviour within this reproducible workflow of calculations, which is normally applied throughout the peptide chain. That is, depending on the specific position of glycines in the chain, in many cases the conventional calculations are not suited to obtaining reliable values of dihedral angles, the estimation of which requires to adopt an alternative equation, specifically tailored to fulfil the specific case. For instance, the conventional procedure used for all residues can be applied to glycine if the amino acid is located in positions 1 or 2 of a peptide sequence. In contrast, if the glycine appears in a different position within the chain, the newly designed Equation (1) is employed (for GLY in position n, with n ≠ 0, 1 and the sign ± in agreement with the sign of φ°_n−1_):

δφ°_GLY_ = (± 3) ∙ (φ°_n−1_) ∙ (δφ°_tc_/180°) n ≠ 1, 2 (1)

In Equation (1), φ°_n−1_ is the total dihedral angle at position n − 1 with respect to a GLY in position n, whereas δφ°_tc_ is the partial dihedral angle arising from the postulated and constant electrostatic interaction between the two terminal charges (i.e., charges on the first and last residue).

This equation does not apply when GLY is found at position n = 1 or 2, since in the case of n = 1 the dihedral angle to calculate is ψ_1_ (being φ_1_ = 0°). For this angle, the same rules considered as for the calculation of the dihedral angles φ [1,2,3,4] are valid and apply consistently. In contrast, if the GLY residue is located in position n = 2, it is clearly not possible to apply Equation (1), due to φ_n−1_ = 0°. Additionally, the methodology of the calculation of dihedral angles ψ has required the introduction of suitable modifications concerning Equation (2), as in “Equation (9)” and as established previously [4], normally applied to obtain ψ values demonstrated not applicable for some residues.

ψ_n_ = ± f · [(φ_n_ · φ_n+1_)/(φ_n_ + φ_n+1_)] ± (× 180°) n ≠ 1 (2)

In particular, we have determined that the value of “f” (the constant required in the prior Equation (9) to calculate the dihedral angles ψ) needs to be changed in specific cases in order to achieve a better match with the experimentally determined dihedral angle values. This may be indicative of differences between small and relatively longer peptide chains that need to be taken into consideration, in order to obtain robust outputs from the prediction analysis. Details concerning the changes about the values of the constant “f” (adjusted values of f concerning chignolin are f = −1/2 for THR6 and f = 1/5 for GLY7, whereas Leu-enkephalin concerns GLY2, GLY3 and PHE4, which have, respectively, f = 3/4, −1/10 and −1/10. In the case of CCR5 ECL2, f = 3/2 for PHE193 and 1/2 for GLN194) are reported in the calculation spreadsheets within the Supporting Information.

**Figure 3:**
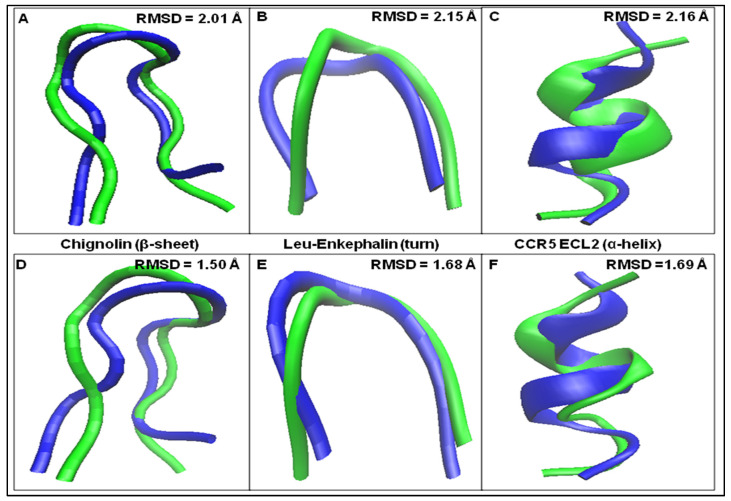
(**A**) Superimposition of the calculated without energy minimization (green) and the experimental structure (blue) of chignolin; (**B**) superimposition of the calculated without energy minimization (green) and the experimental structure (blue) of Leu-enkephalin; (**C**) superimposition of the calculated without energy minimization (green) and the experimental structure (blue) of CCR5 ECL2; (**D**) superimposition of the calculated after energy minimization (green) and the experimental structure (blue) of chignolin; (**E**) superimposition of the calculated after energy minimization (green) and the experimental structure (blue) of Leu-enkephalin; and (**F**) superimposition of the calculated after energy minimization (green) and the experimental structure (blue) of CCR5 ECL2.

By adopting these modifications and applying the workflow previously developed [4] for the calculation of dihedral angles in peptides, we have analysed herein chignolin, Leu-enkephalin and CCR5 ECL2. Each dihedral angle in these polypeptides was calculated. The final structures were modelled in VMD [14,15] and compared by superimpositions to those experimentally available. Overall, a robust match in terms of RMSD was obtained throughout the analysis. By submitting the calculated structures to energy minimization using the web server Chiron [16], an RMSD improvement was achieved, as evident from the decrease of all RMSD values, which in each case are below 1.70 Å. Details of these results are shown in Figure 3, where the superimposed structures A–C are those without energy minimization, whereas D–F are those after energy minimization.

**Figure 4:**
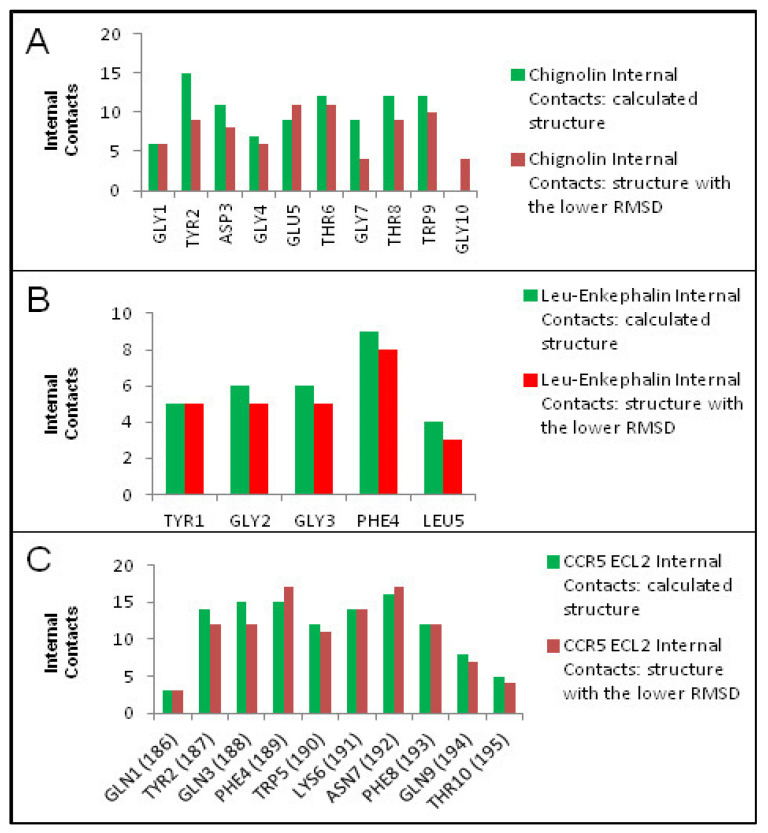
Per-residue internal contacts between the calculated/predicted structures (green) and the structures with the lowest RMSD during MD simulations (red). (**A**) Chignolin, (**B**) Leu-enkephalin and (**C**) CCR5 ECL2.

Moreover, we have submitted the three oligopeptides to molecular dynamics (MD) simulations. The MD simulations of the complexes were performed with YASARA. A periodic simulation cell extending 10 Å from the protein surfaces were employed. The cell was filled with water, with a maximum sum of all water bumps of 1.0 Å and a density of 0.997 g/mL. The setup included optimizing the hydrogen bonding network [17] to increase the solute stability and a pKa prediction to fine-tune the protonation states of protein residues at the chosen pH of 7.4 [18]. With an excess of either Na or Cl to neutralize the cell, NaCl ions were supplied at a physiological concentration of 0.9%. The cutoff was 10 Å for Van der Waals forces (the default used by AMBER) [19], and no cutoff was applied to electrostatic forces (using the Particle Mesh Ewald algorithm) [20]. The equations of motions were integrated with multiple time steps of 2.5 fs for bonded interactions and 5.0 fs for nonbonded interactions at a temperature of 298 K and a pressure of 1 atm. Initially, a short MD simulation was run on the solvent only to remove clashes. The entire system was then energy minimized using a steepest descent minimization to remove conformational stress, followed by a simulated annealing minimization until convergence (<0.01 kcal/mol Å). Finally, 100 ns MD simulation without any restrictions was conducted and the conformations were recorded every 200 ps (details on the MD simulations can be accessed via the Supporting Information file, Figures S1–S18). We have subsequently compared the structures obtained by calculations with the corresponding ones having the lower RMSD from MD simulations in water (1.81 Å for chignolin, 1.43 Å for Leu-Enkephalin and 1.2 Å for CCR5 ECL2). The comparison was performed to show how the internal contacts of each residue are determined (method reported in Supporting Information, Supplementary Materials File S1). Substantially, we found that between the calculated oligopeptides and the corresponding structures with the lowest values of RMSD in MD simulations, the number of internal contacts is comparable, except for GLY10 in chignolin (Figure 4). We explain this output as a confirmation of the fact that the hydrophobic effect arising after solubilisation in water, proceeds by maximizing the internal contacts, allowing to establish the interactions of interest that drive the folding process of all the three oligopeptides investigated in this work.

## 3. Discussion and Conclusions

To conclude, the MMFs approach demonstrated a valid tool to analyse the folding mechanism of the oligopeptides selected for this study, enabling us to obtain information at the single-residue level. To this end, we propose the adoption of “single residues cooperative folding” as a suitable term to define the folding mechanism under the circumstances assessed in this work. Clearly, we have established that certain type of interactions, namely hydrophobic and CH–π cloud, dominate over others, such as H-bonds and electrostatic attraction/repulsion. Hydrophobic and CH–π cloud interactions were thus considered as the main output of the hydrophobic effect, while H-bonds and inter-residue electrostatic interplays are likely playing a role in stabilizing the peptide structures. It is noteworthy that hydrophobic and CH–π cloud interactions confer the largest rotations in terms of partial dihedral angles and result in the most frequent types of interplays that provide a better match with the experimental data. The outcomes of this study support widely earlier findings [5,21], indicating that the folding is the result of a “cooperative activity” within amino acid residues. The process might be influenced and, primarily, proceed through the interplay amongst apolar or “hydrophobic” amino acids, although larger and more polar side chains (e.g., methionine or, alternatively, charged groups as in lysine and arginine) may also play a role in particular circumstances concerning the dominant interactions pointed out in this study.

## Data Availability

The original contributions presented in the study are included in the article/Supplementary Materials, further inquiries can be directed to the corresponding authors.

## Supplementary Materials

The following supporting information can be downloaded at: https://www.mdpi.com/article/10.3390/ijms25179586/s1.
