# Supplementary material for: Dominant Chemical Interactions Governing the Folding Mechanism of Oligopeptides
Source: Int J Mol Sci. 2024 Sep 4;25(17):9586. doi: 10.3390/ijms25179586 (PMC11395422; doi:10.3390/ijms25179586)
Supplement: Supplementary file 1 [file ijms-25-09586-s001.zip › Nuova cartella/Supplementary Materials File S1.pdf]

Supporting information

Dominant Chemical Interactions Governing the Folding Mechanism of Oligopeptides

Michele Larocca [1], Giuseppe Floresta,[2] Daniele Verderese [3] and Agostino Cilibrizzi* [4,5]*

[1]Istituto di Metodologie per l'Analisi Ambientale – Consiglio Nazionale delle Ricerche (CNR-IMAA), C.da S. Loja, 85050 Tito Scalo (PZ), Italy.

[2]Department of Drug and Health Sciences, University of Catania, Viale A. Doria 6, 95125 Catania, Italy

[3]Dipartimento di Scienze Economiche e Statistiche, Università di Salerno, via Giovanni Paolo II, 132, 84084 Fisciano (SA), Italy

[4]Institute of Pharmaceutical Science, King's College London, Stamford Street, London SE1 9NH, UK

[5]Centre for Therapeutic Innovation, University of Bath, Bath BA2 7AY, UK

Content:

1. **Determination of the internal contacts**
2. **Figures S1-S18**. Analyses inside the simulation cell for each MD simulation

1. Determination of the internal contacts

To determine the internal contacts of each oligopeptide, a specific VMD plug-in was employed and set as it follows:

- Open VMD and select the ''Hydrogen Bonds'' plug-in;
- Select ''Both'' in Selection 1 is the:;
- Set a Donor-Acceptor distance of 4.0 Å;
- Set an Angle cut-off of 180°;
- Select ''Residue Pairs'' in Calculate detailed info for:;
- Press ''Find hydrogen bonds!''.

2. Analyses inside the simulation cell for each MD simulation

CHIGNOLIN – PDB code: 1UAO

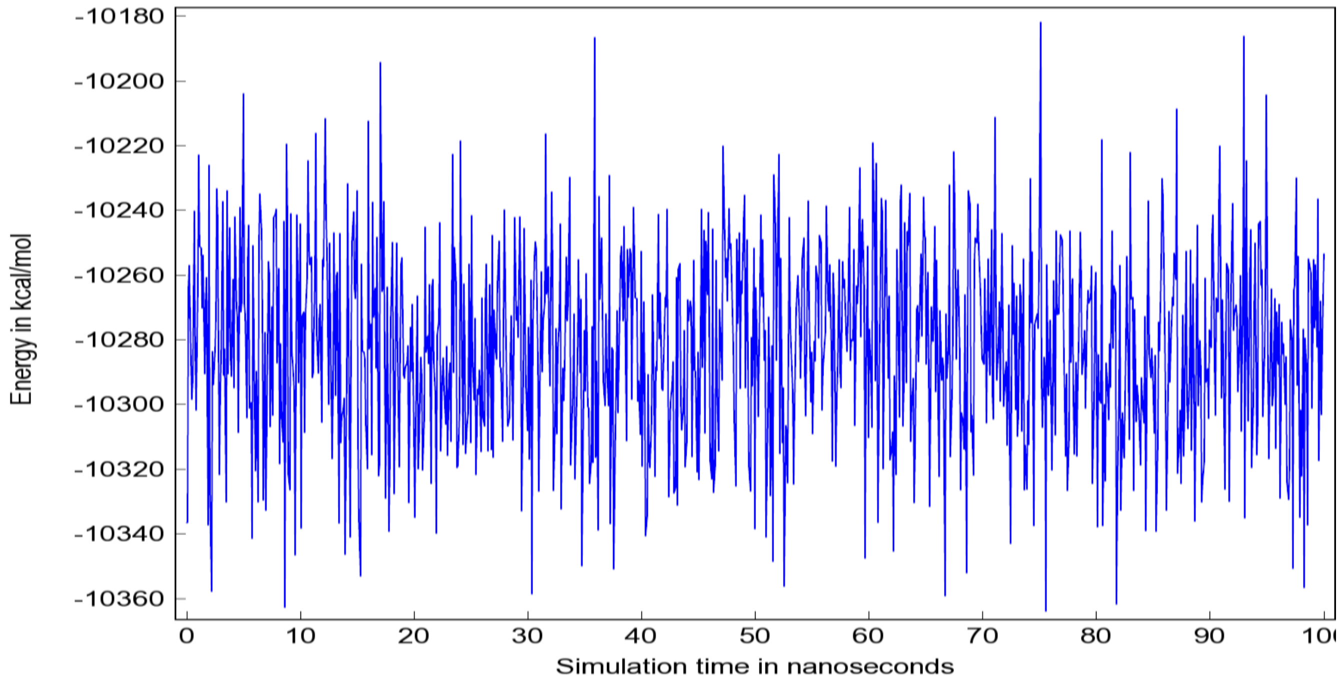

Figure S1. Chignolin: total potential energy of the system.

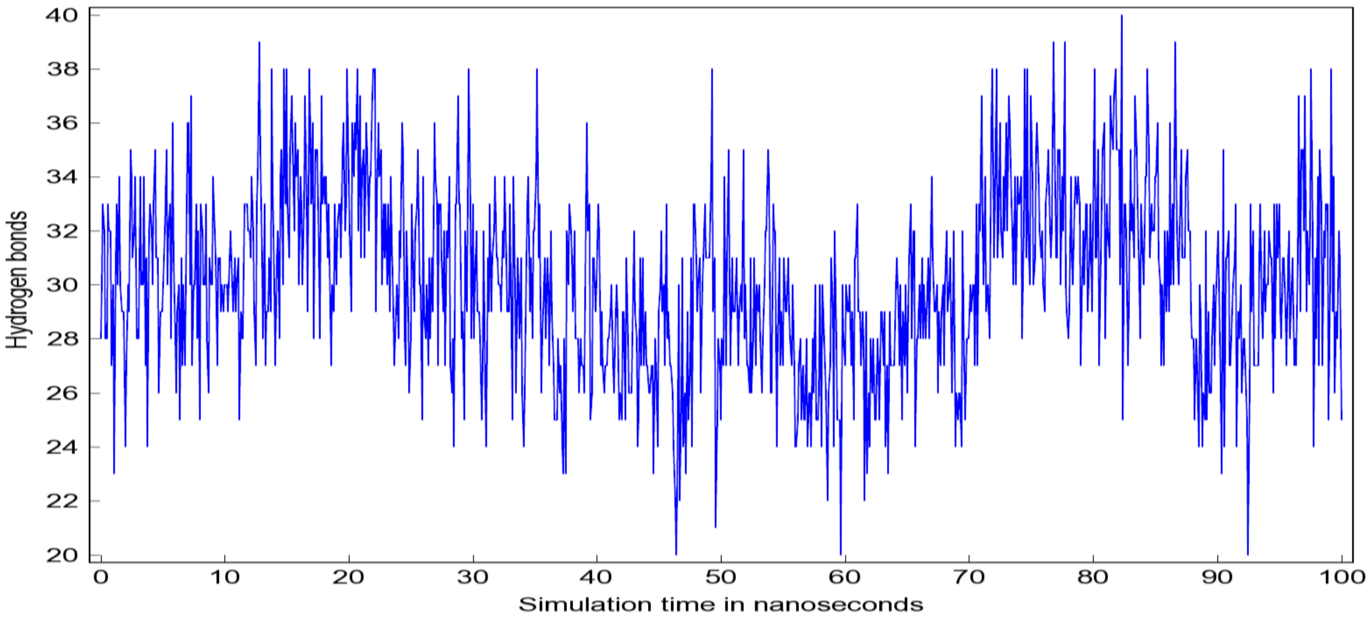

Figure S2. Chignolin: Number of hydrogen bonds between solute and solvent.

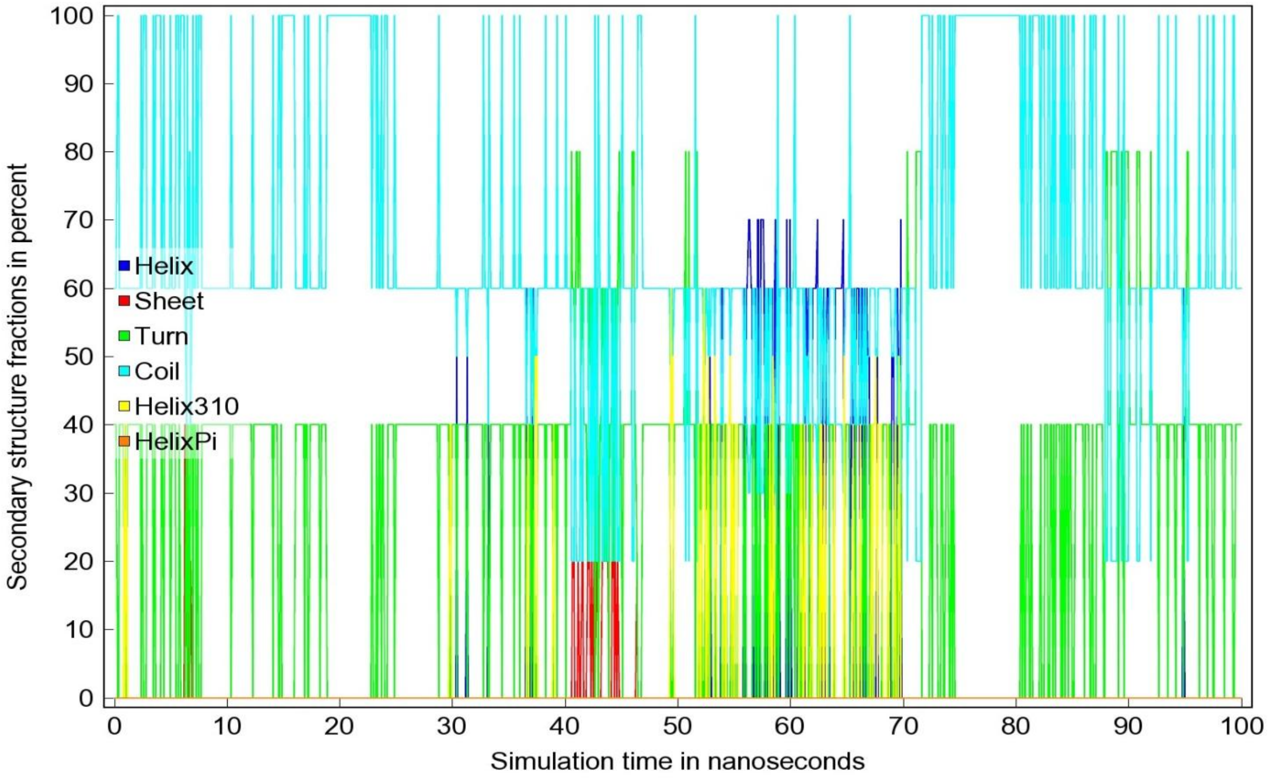

Figure S3. Secondary structure content of Chignolin.

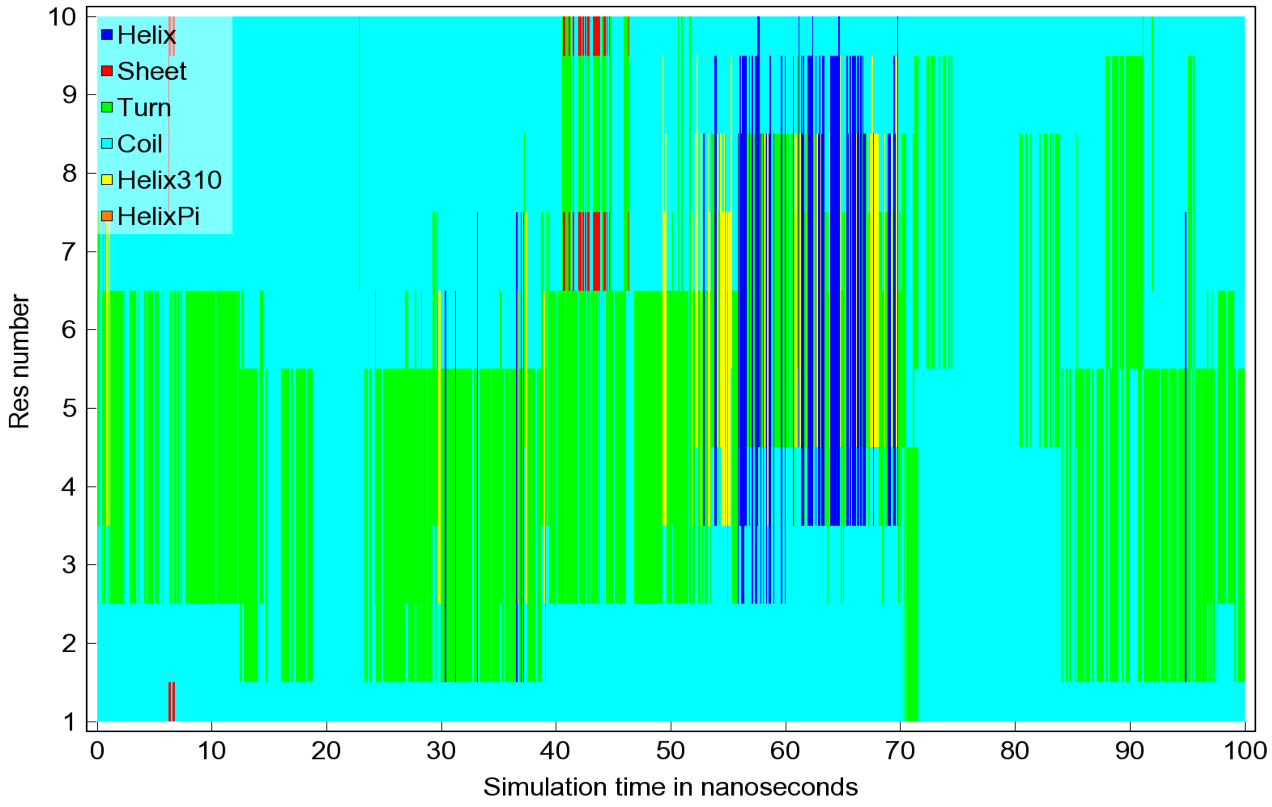

Figure S4. Per-residue secondary structure of Chignolin.

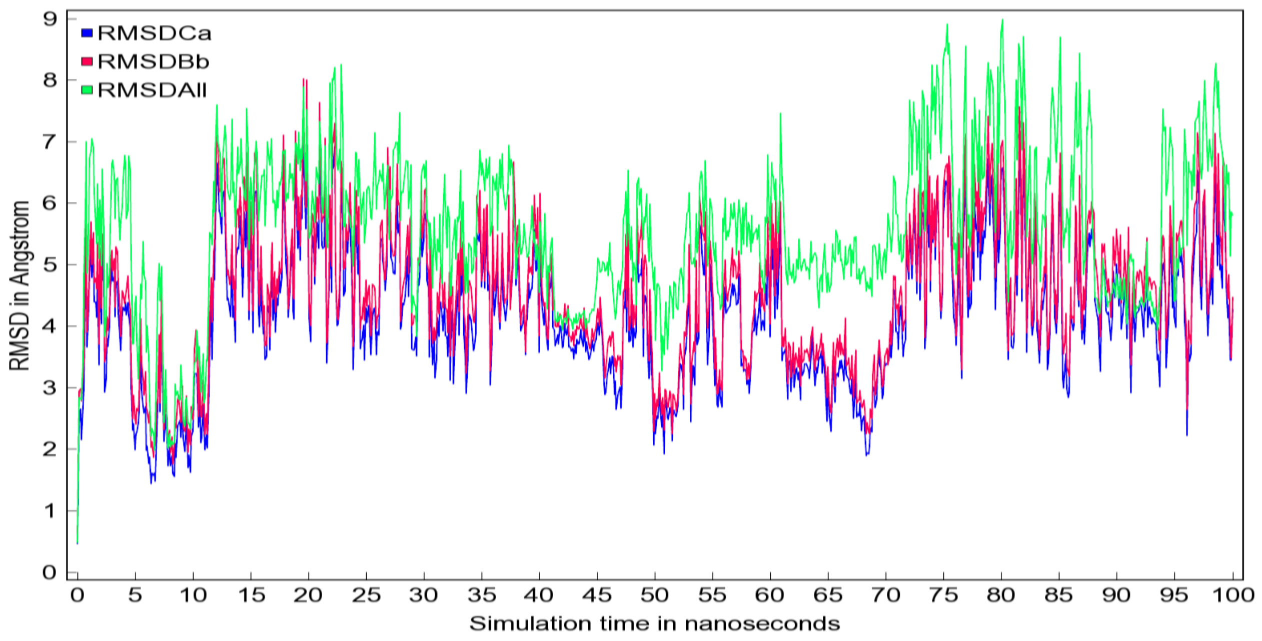

Figure S5. Chignolin: solute RMSD from the starting structure.

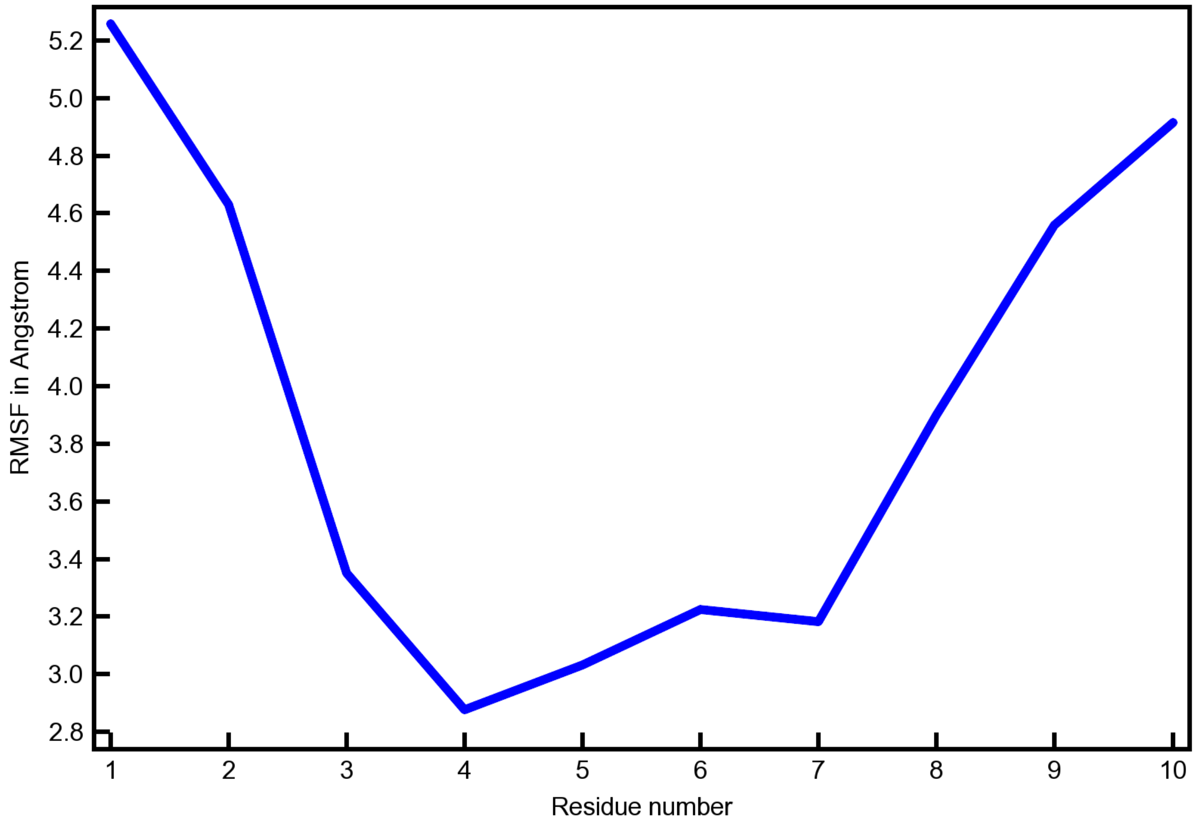

Figure S6. Chignolin: solute residue RMSF.

LEU-ENKEPHALIN – PDB code: not available

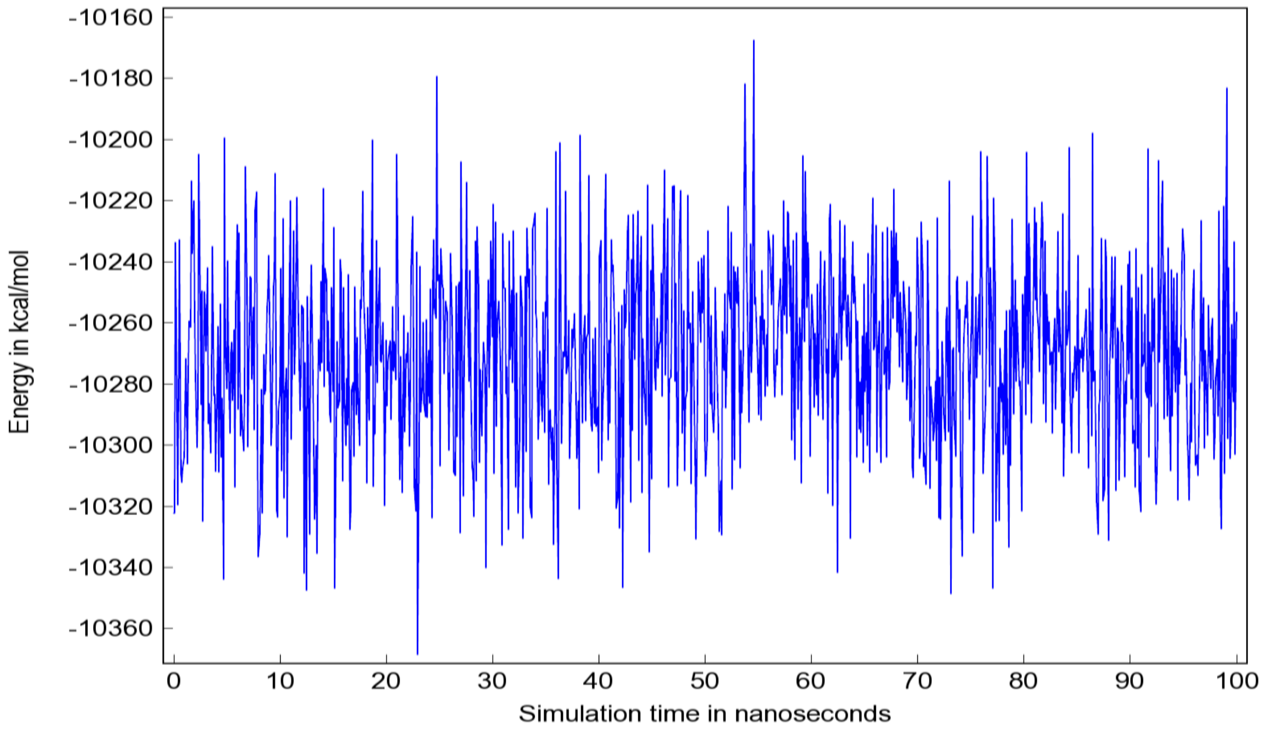

Figure S7. Leu-enkephalin: total potential energy of the system.

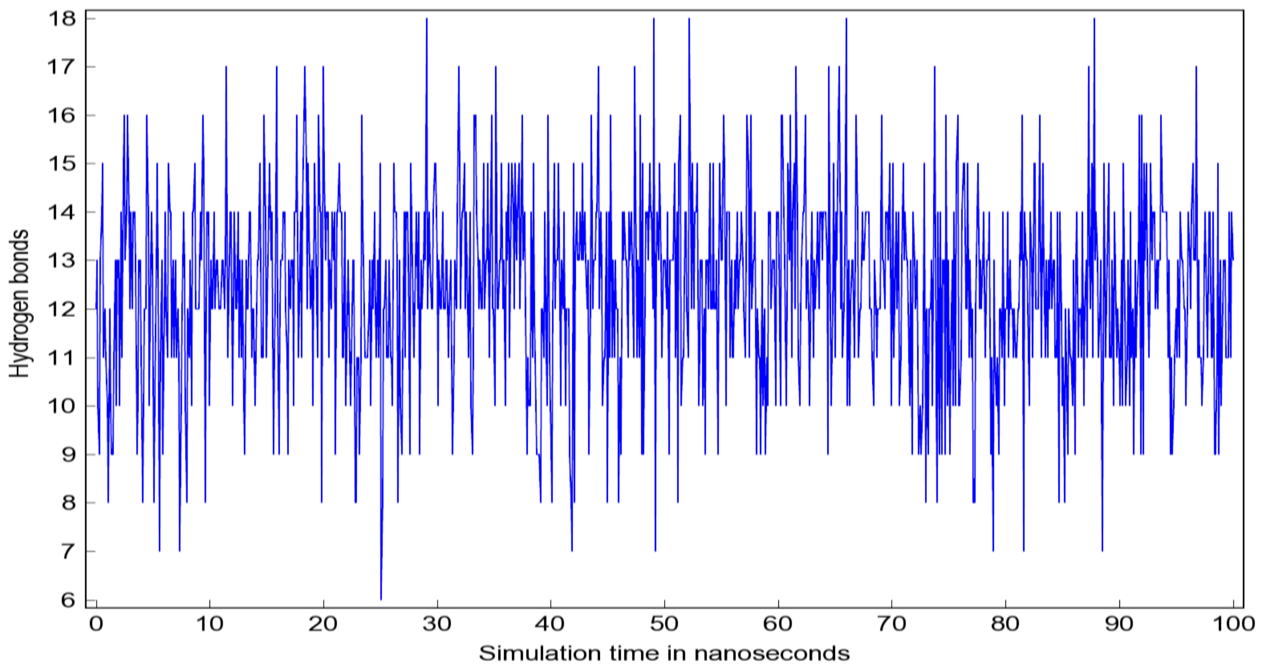

Figure S8. Leu-enkephalin: Number of hydrogen bonds between solute and solvent.

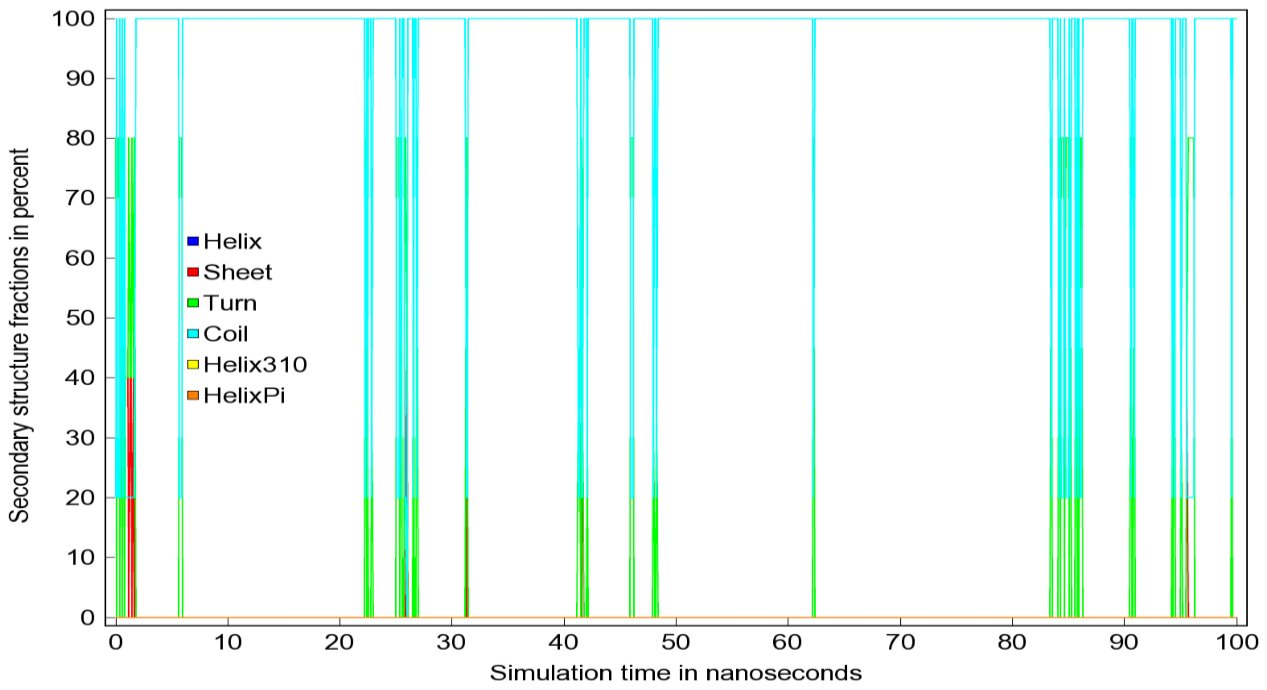

Figure S9. Secondary structure content of Leu-enkephalin.

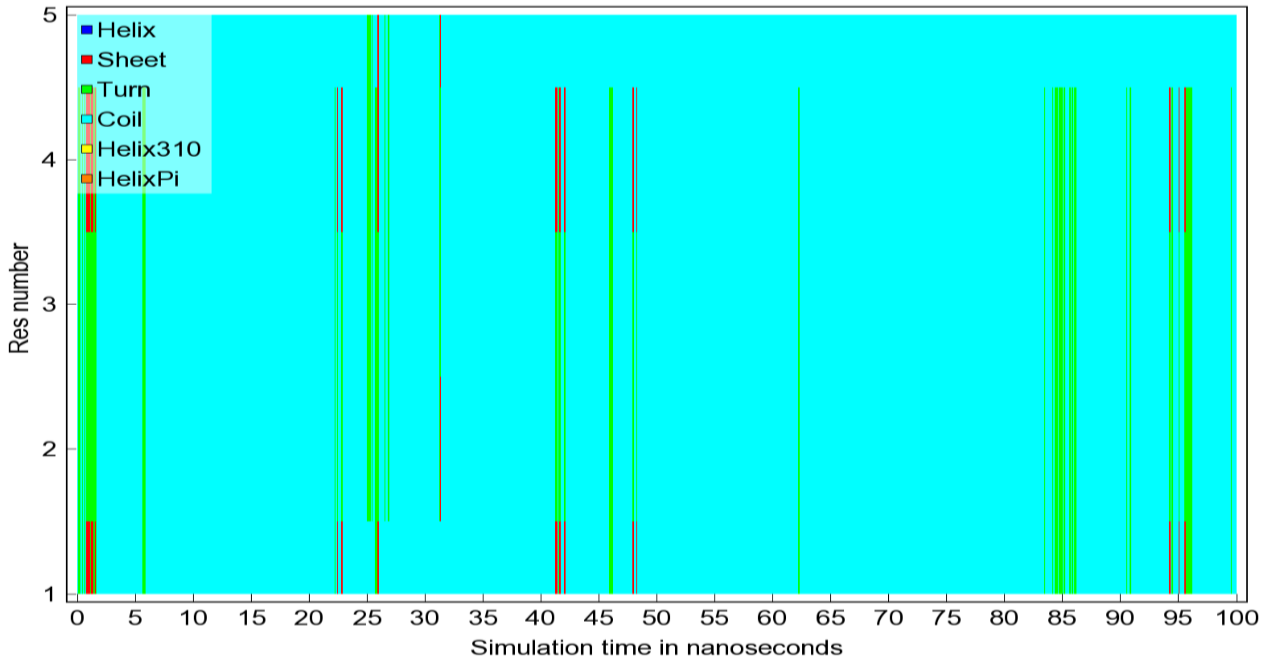

Figure S10. Per-residue secondary structure of Leu-enkephalin.

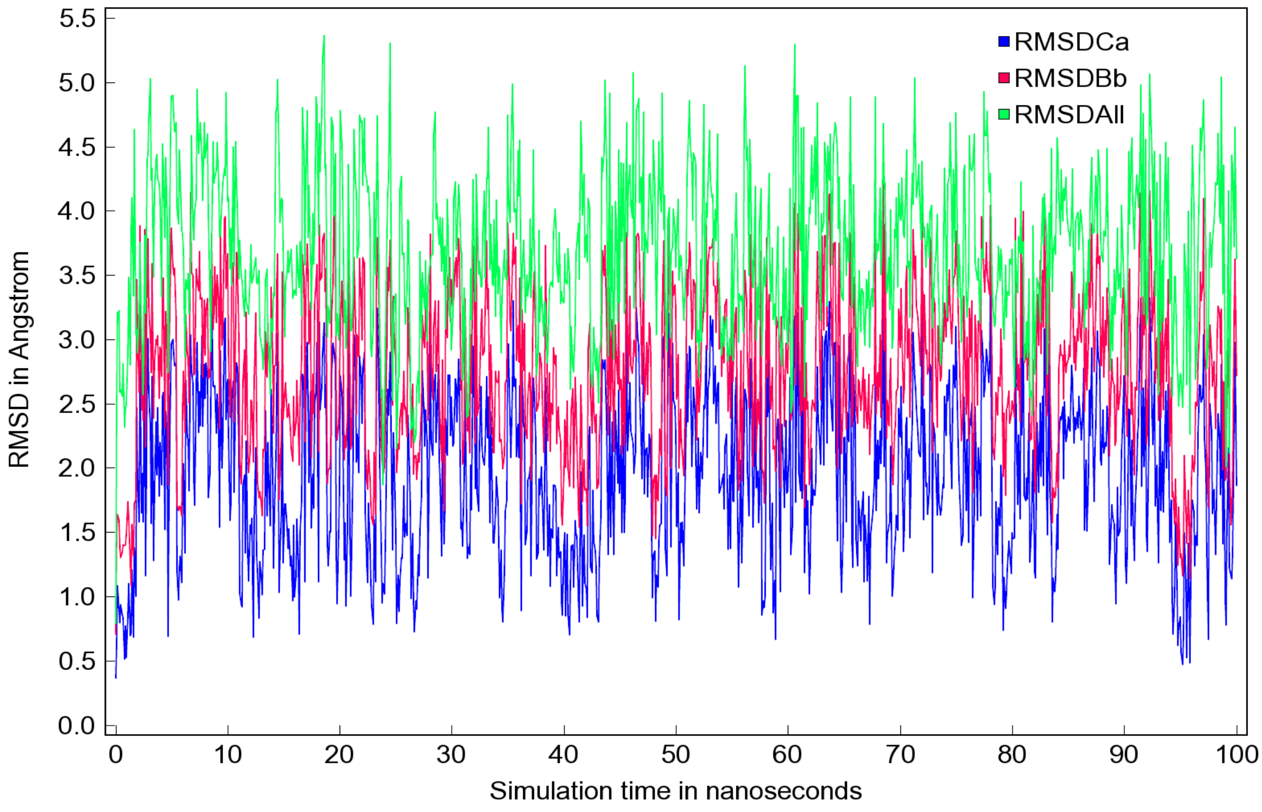

Figure S11. Leu-enkephalin: solute RMSD from the starting structure.

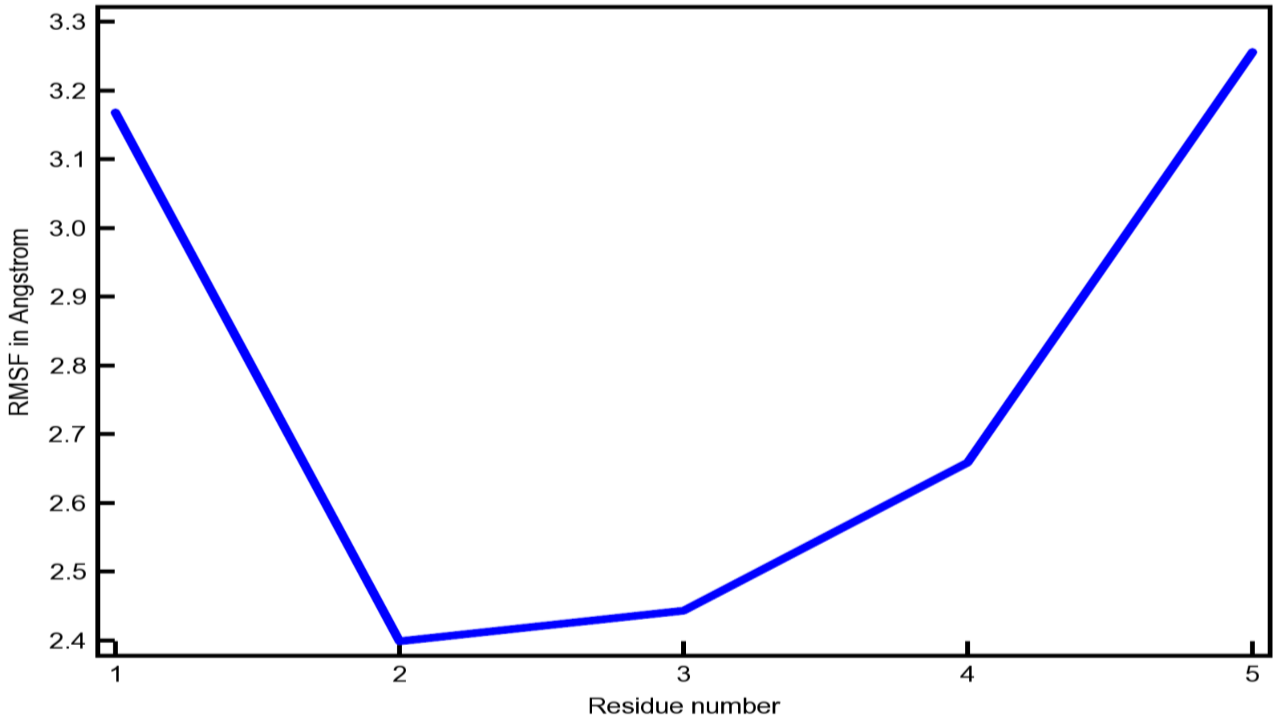

Figure S12. Leu-enkephalin: solute residue RMSF.

CCR5 ECL2 – PDB code: 2MZX

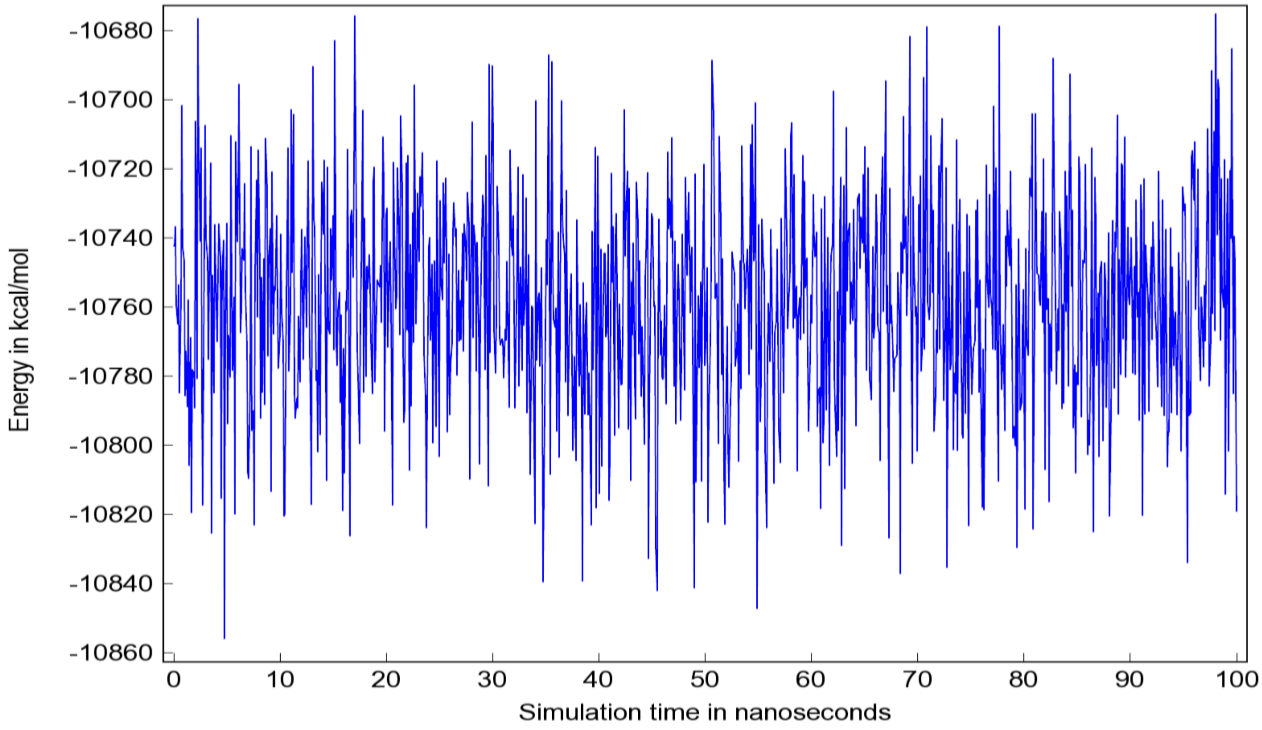

Figure S13. CCR5 ECL2: total potential energy of the system.

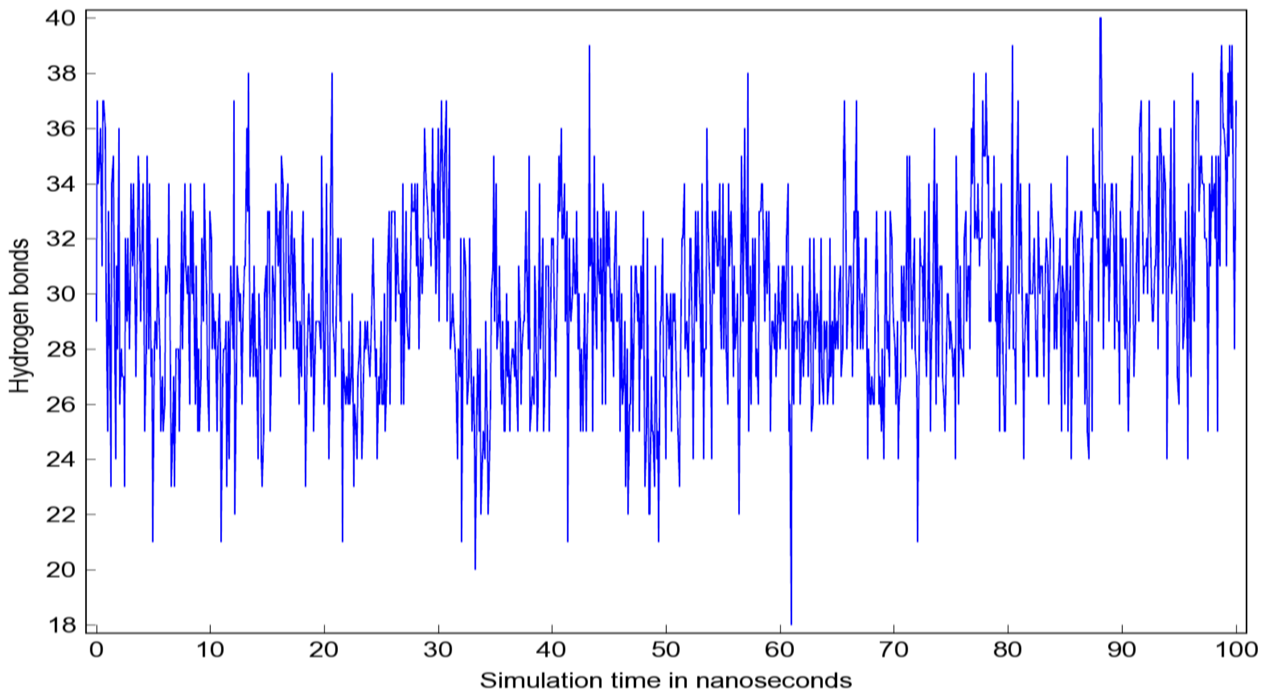

Figure S14. CCR5 ECL2: Number of hydrogen bonds between solute and solvent.

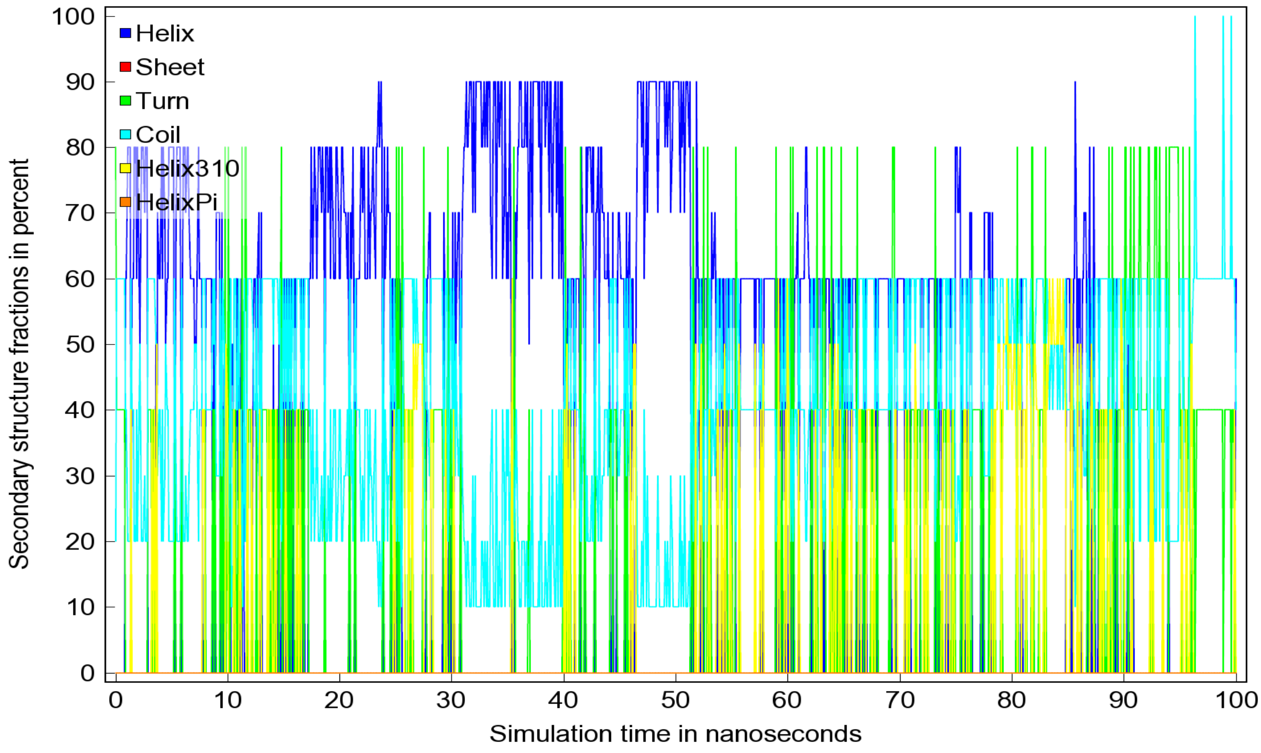

Figure S15. Secondary structure content of CCR5 ECL2.

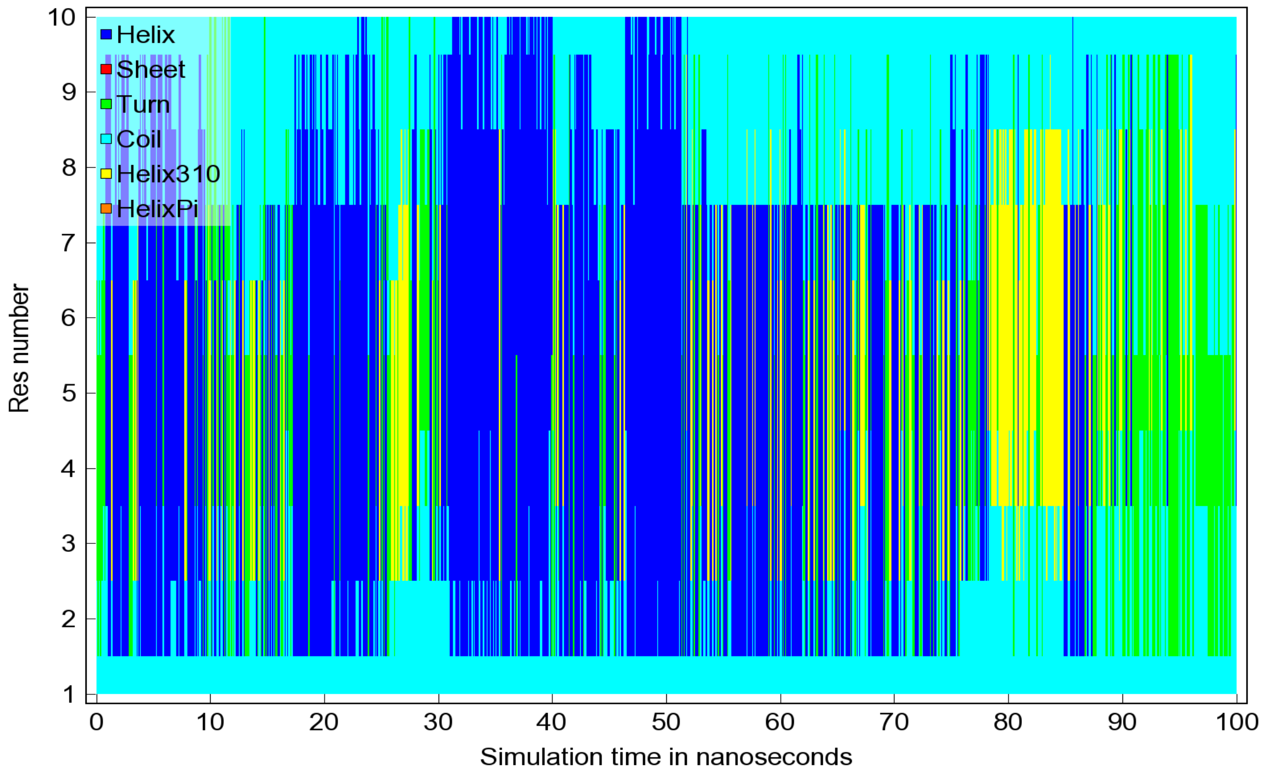

Figure S16. Per-residue secondary structure of CCR5 ECL2.

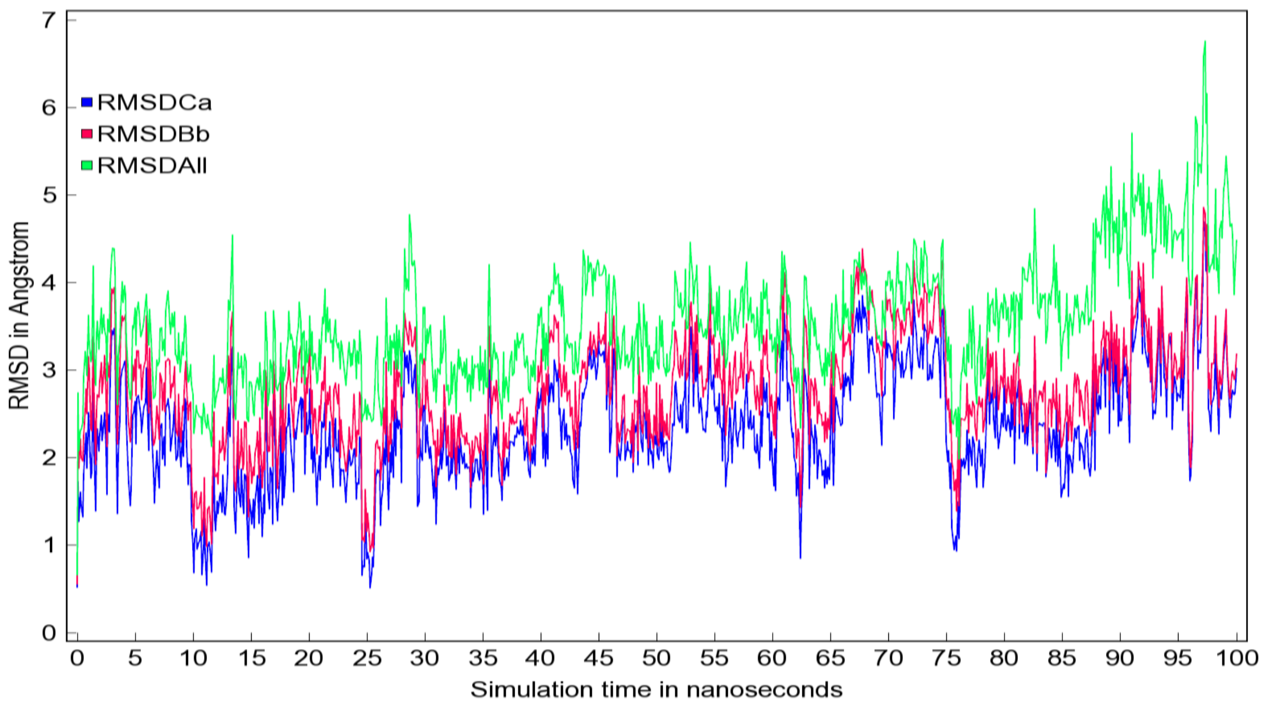

Figure S17. CCR5 ECL2: solute RMSD from the starting structure.

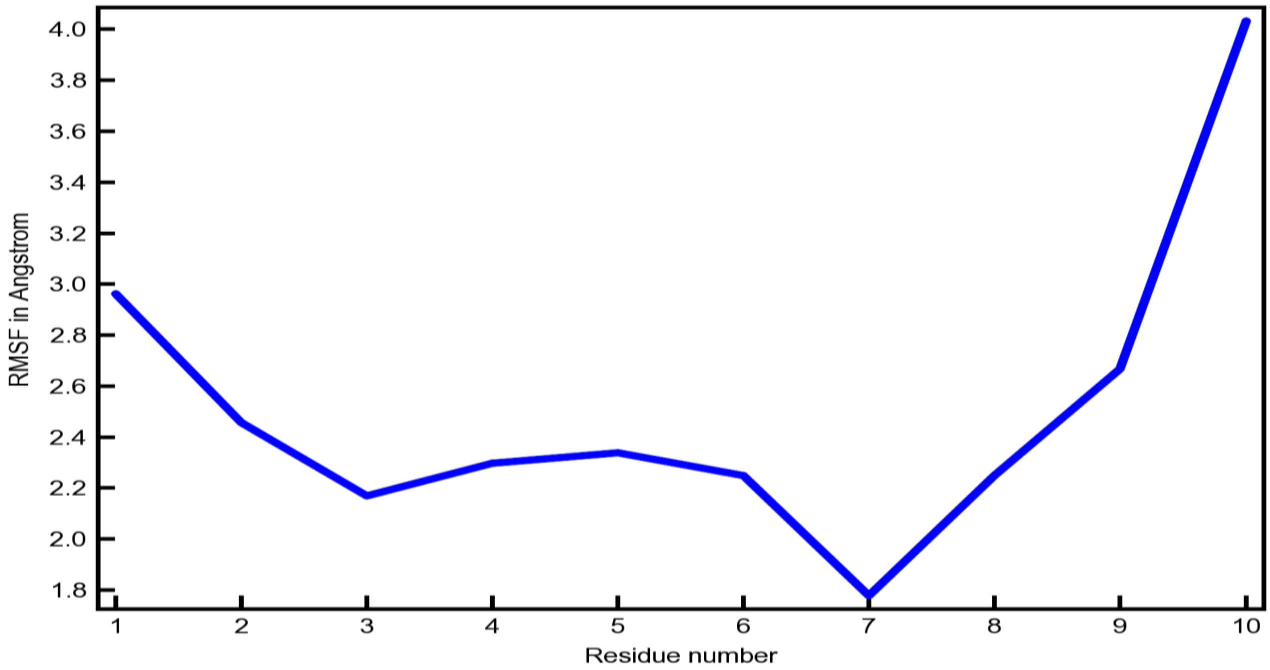

Figure S18. CCR5 ECL2: solute residue RMSF.
